# Exploring the Molecular Mechanism of Hepatic Dysfunction Among Workers Exposed to Nickel and Chromium in Electroplating

**DOI:** 10.3390/ijms262411954

**Published:** 2025-12-11

**Authors:** Mona Abdallah Ramadan, Marwa Abdelgwad, Reem T. Atawia, Amira M. Badr, Eman Mahmoud Khalifa, Layla A. Alkharashi, Rateba Said Mohammed

**Affiliations:** 1 Occupational and Environmental Medicine Department, Faculty of Medicine, Cairo University, Al Giza 12613, Egypt; 2 Medical Biochemistry and Molecular Biology Department, Faculty of Medicine, Cairo University, Al Giza 12613, Egypt; 3 Department of Pharmaceutical Sciences, College of Pharmacy, Southwestern Oklahoma State University, Weatherford, OK 73096, USA; 4 Department of Pharmacology and Toxicology, Faculty of Pharmacy, Ain Shams University, Cairo 11566, Egypt; 5 Department of Pharmacology and Toxicology, College of Pharmacy, King Saud University, P.O. Box 2457, Riyadh 11415, Saudi Arabia; amibadr@ksu.edu.sa (A.M.B.);

**Keywords:** hepatotoxicity, chromium, nickel, electroplating workers, oxidative stress, *Keap-1*/*Nrf2*

## Abstract

Exposure to nickel (Ni) and chromium (Cr) in environmental and occupational settings appears to be inevitable and significantly affects the liver, the principal organ responsible for their metabolic processes. This research aimed to assess the functional integrity of the liver and the molecular mechanisms underlying hepatic damage in employees exposed to Ni and Cr at work. A cross-sectional investigation was implemented with 86 non-smoking male employees working in a metallurgical factory. Serum Cr, Ni, liver function tests, oxidative and inflammatory indicators, and *Keap-1*, *Nrf2*, and *miR-223* expression were assessed. In electroplating workers, serum Cr (2.47 ± 2 µg/L), Ni (1.39 ± 0.79 µg/L), liver transaminases, total bilirubin, and NF-κB were all statistically significantly greater than in the referent group. Electroplaters’ serum albumin levels were significantly lower than those of controls. Furthermore, oxidative stress was observed in electroplaters, characterized by lower levels of superoxide dismutase (SOD) and glutathione peroxidase (GPx) and greater levels of malondialdehyde (MDA) with respect to controls (*p* < 0.05). Additionally, compared to controls, gene expressions in electroplaters showed that *Keap-1* was upregulated, while *Nrf2*/*Ho-1* and *miR-223* were downregulated. In conclusion, occupational exposure to Ni and Cr was associated with hepatic impairment through downregulation of the antioxidant *Nrf2* pathway, oxidative stress, and inflammation.

## 1. Introduction

Electroplating is a critical stage in the metal-finishing process in numerous sectors. Augmenting the hardness, abrasion, and corrosion resistance of the metals utilized generally enhances their mechanical and chemical properties. Workers who perform electroplating are exposed to chromic acid in its hexavalent form, which is utilized as an electrolyte-forming mist during the plating process. Additionally, exposure occurs during the weighing and mixing of acid powders and while transferring liquids and objects into and out of plating baths [1]. Since nickel (Ni) plating is a common component of chromium (Cr) plating, chrome platers are frequently exposed to Ni compounds, such as powders and mists of Ni sulfate and Ni chloride [2].

Workers may be contaminated with hexavalent Cr and Ni mostly by inhalation and dermal contact, allowing these substances to enter the circulatory system and be subsequently metabolized by the liver [3]. The buildup of these metals may harm the liver, an essential organ engaged in the metabolism and transformative processes of Cr and Ni [4]. Evidence regarding the correlation between Cr exposure and hepatotoxicity is expanding [5]. Metallic contaminants have a particularly detrimental impact on the liver; the liver is substantially impacted by exposure to Ni and Cr [6,7].

Oxidative stress is a key molecular and cellular mechanism linked to the pathophysiology of injuries to the liver associated with Ni and Cr exposure, significantly contributing to the initiation and advancement of liver illnesses. When the ratio of Reactive Oxygen Species (ROS) to antioxidant defenses is disproportionately weighted towards pro-oxidant substances, this deleterious sequence of ROS damages proteins, lipids, and Deoxyribonucleic Acid (DNA) [8]. The liver is strongly guarded against oxidative damage, mediated by the nuclear factor-erythroid 2-related factor 2 (Nrf2) response. Nrf2 is intricately linked to kelch-like Enoyl-CoA Hydratase (ECH)-associated protein 1 (*Keap-1*) and governs the expression of phase II detoxifying enzyme genes and antioxidant response genes [9]. According to Han and colleagues, numerous hazardous reactions linked to Cr exposure are significantly influenced by the Nrf2 system [10].

Additionally, systemic inflammation is considered a critical factor in metal-induced liver disorders, as the metal generates ROS that induce systemic inflammation [11]. Prior studies have shown that exposure to Cr and Ni induces an inflammatory response via the activation of the NF-κB pathway. This pathway is crucial for the onset and advancement of inflammation and plays a significant role in the transcription of many pro-inflammatory proteins that induce liver damage [6,12].

Moreover, microRNAs (miRNAs) are short, non-coding RNAs that inhibit gene expression at the post-transcriptional stage by attaching to the 3-untranslated regions (3-UTR) of target mRNAs [13]. Several clinical investigations have indicated that miRNA dysregulation may have a substantial role in the development of severe liver-related conditions, including infectious viral hepatitis, fibrosis of the liver, acute liver conditions, drug-induced liver injury (DILI), metabolic problems with the liver, and hepatocellular carcinoma (HCC) [14]. Recent research suggests that miR-223 plays an essential role in the immune system’s development and homeostasis and may play a fundamental role in oxidative stress and inflammatory disorders by targeting the *Keap-1*/Nrf2 cascade that contributes to a variety of liver diseases [15,16]. Recently, it was discovered that miRNA dysregulation plays a significant role in the cytotoxicity, carcinogenesis, and angiogenesis induced by Ni and Cr [17].

Therefore, occupational exposure to Cr and Ni is a growing concern due to the hepatotoxicity it induces. However, the underlying mechanism for this hepatotoxicity has not been thoroughly examined in occupationally exposed workers. Hence, in this study, we hypothesized that exposure of electroplaters to Ni and Cr can be associated with impairment in the functional integrity of the liver, which is mediated by changes in oxidative stress-related genes and *miR-223* expression levels, in addition to the induction of inflammation.

## 2. Results

The study comprised 86 non-smoking participants, including 43 male electroplating industry workers who were matched with 43 control participants from the administrative staff based on age, sex, and body mass index (BMI). Regarding the symptoms, signs, and health risks associated with exposure to Ni and Cr during the electroplating process, electroplating workers had a statistically significantly higher prevalence of irritating effects such as contact dermatitis (18.6%), rhinitis (18.6%), cough (30.2%), expectoration (25.6%), and chronic fatigue (32.6%) compared to the referent group (Table 1).

Electroplating workers had an average occupational exposure time of 18.26 ± 9.58 years (range 6–35). They typically work eight-hour shifts five days a week. Over a third (39.5%) wore heat-resistant and flame-retardant suits, overalls, gloves, safety goggles, face shields, N95 air-purifying respirators, and boots regularly as part of their personal protective equipment (PPE). Regarding blood pressure, BMI, age, and tenure of employment, no statistically significant differences were found between the exposed and control groups (Table 2).

**Figure 1:**
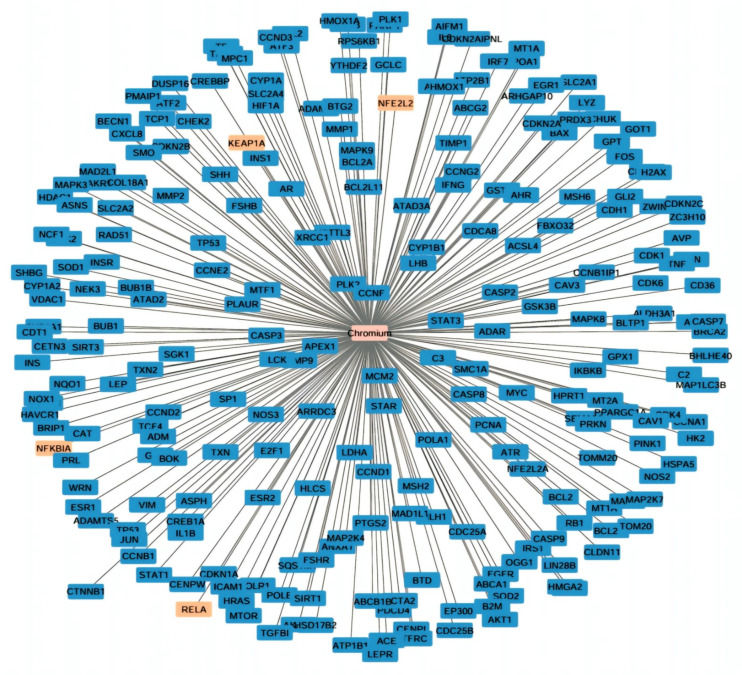
Cystoscope network visualization of chromium-interacting genes. Highlighted genes in orange include *NFE2L2* (*Nrf2*) and its negative regulator *Keap-1* (Kelch-like ECH-associated protein 1), *RELA* (*NF-κB p65* subunit), and *NF-κBIA* (*NF-κB* inhibitor-alpha, IκBa).

The electroplating workers’ mean serum levels of Cr (2.47 ± 2 µg/L), Ni (1.39 ± 0.79 µg/L), liver transaminases (ALT and AST), as well as total bilirubin were all statistically significantly greater than those of the referent group. Additionally, the electroplaters’ serum albumin levels were significantly lower than those of the control group (4.21 ± 0.93 vs. 4.65 ± 0.67, respectively). Electroplating workers had statistically significant lower levels of SOD (372.31 ± 162.44) and GPx (34.73 ± 13.45) and a statistically significant higher level of MDA (15.02 ± 3.99), indicating overwhelming oxidative stress status. Furthermore, a statistically significant difference in the exposed group’s inflammatory marker (NF-κB) level from the referent group was noted (0.46 ± 0.2 vs. 0.26 ± 0.04, respectively) (Table 2). Also, using the Comparative Toxigenomics Database to determine genes interacting with chromium. Interestingly, *NFE2L2* (*Nrf2*) and its negative regulator *Keap-1* (Kelch-like ECH-associated protein 1), *RELA* (*NF-κB p65* subunit), and *NF-κBIA* (*NF-κB* inhibitor-alpha, IκBa) are among chromium-interacting genes (Figure 1).

Our analysis of the oxidative stress and inflammatory gene expression of electroplaters revealed that, compared to controls, the *Nrf2, Ho-1*, and *miR-223* gene expressions were statistically significantly downregulated. In contrast, electroplating workers showed significant upregulation of the *Keap-1* gene (Table 2).

Regarding the relationship between the usage of PPE among electroplaters and the levels of measured biochemical markers, electroplaters who regularly used their PPE showed a significantly lower level of Cr, Ni, hepatic transaminases, and MDA (Table 3).

**Figure 2:**
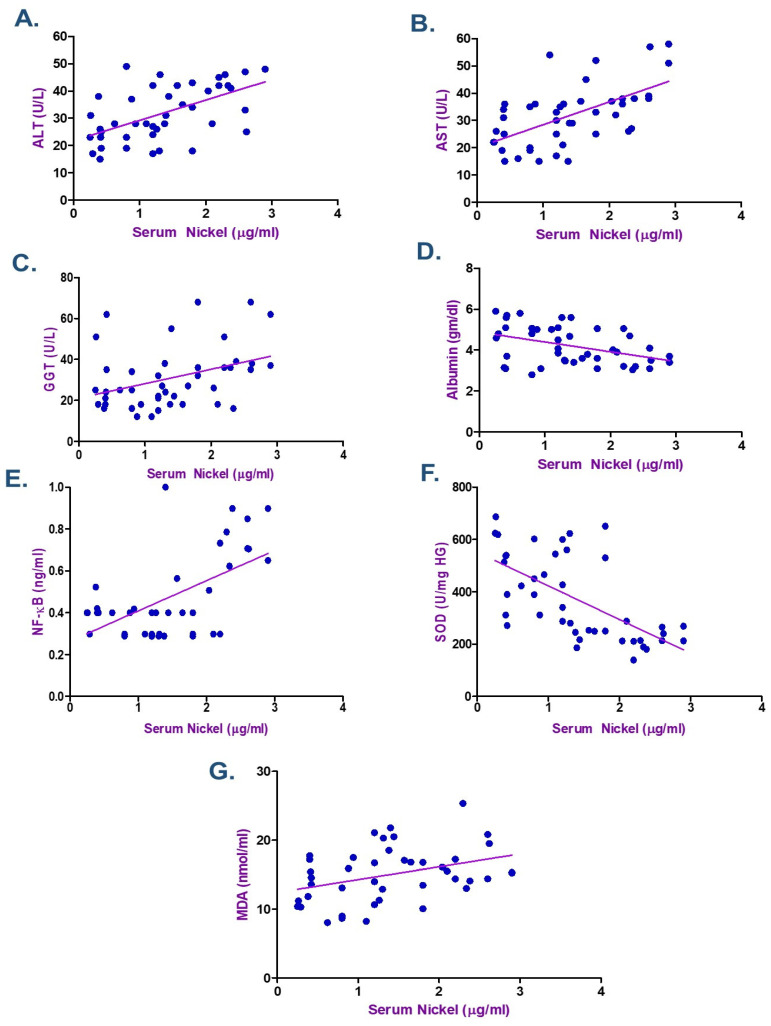
Spearman Correlation between Serum Nickel and (**A**) Alanine transaminase (ALT), (**B**) Aspartate transaminase (AST), (**C**) γ-Glutamyl transferase (GGT) (**D**) Albumin, (**E**) Nuclear factor-κB (NF-κB), (**F**) Superoxide dismutase (SOD), and (**G**) Malondialdehyde (MDA), among Electroplating Workers (the line represents the fitted correlation trend, and the dots indicate the individual data points used to calculate the correlation coefficient).

**Figure 3:**
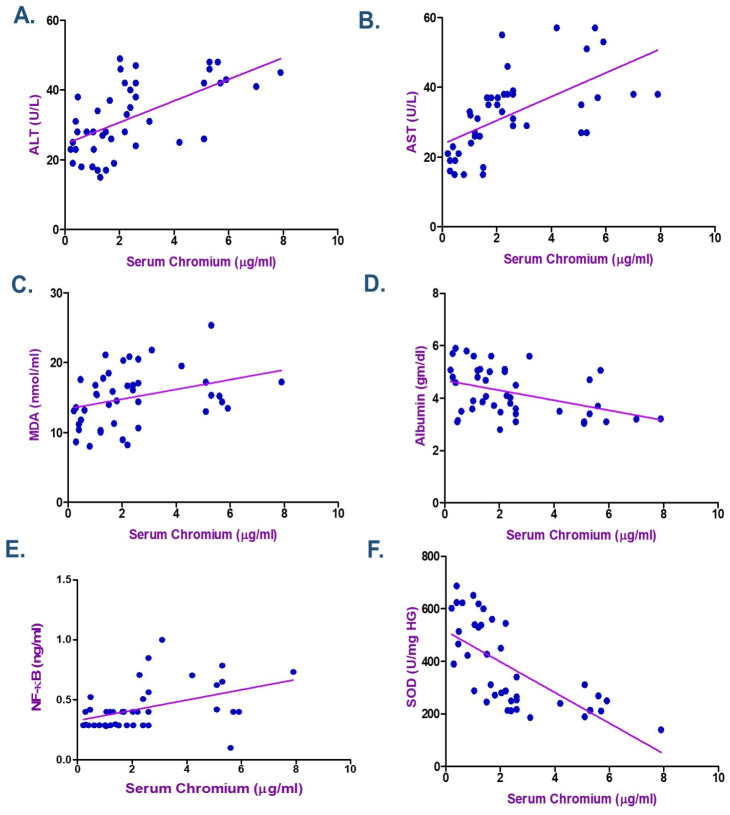
Spearman Correlation between Serum Chromium and (**A**) Alanine transaminase (ALT), (**B**) Aspartate transaminase (AST), (**C**) Malondialdehyde (MDA), (**D**) Albumin, (**E**) Nuclear factor-κB (NF-κB), and (**F**) Superoxide dismutase (SOD) among Electroplating Workers (the line represents the fitted correlation trend, and the dots indicate the individual data points used to calculate the correlation coefficient).

**Figure 4:**
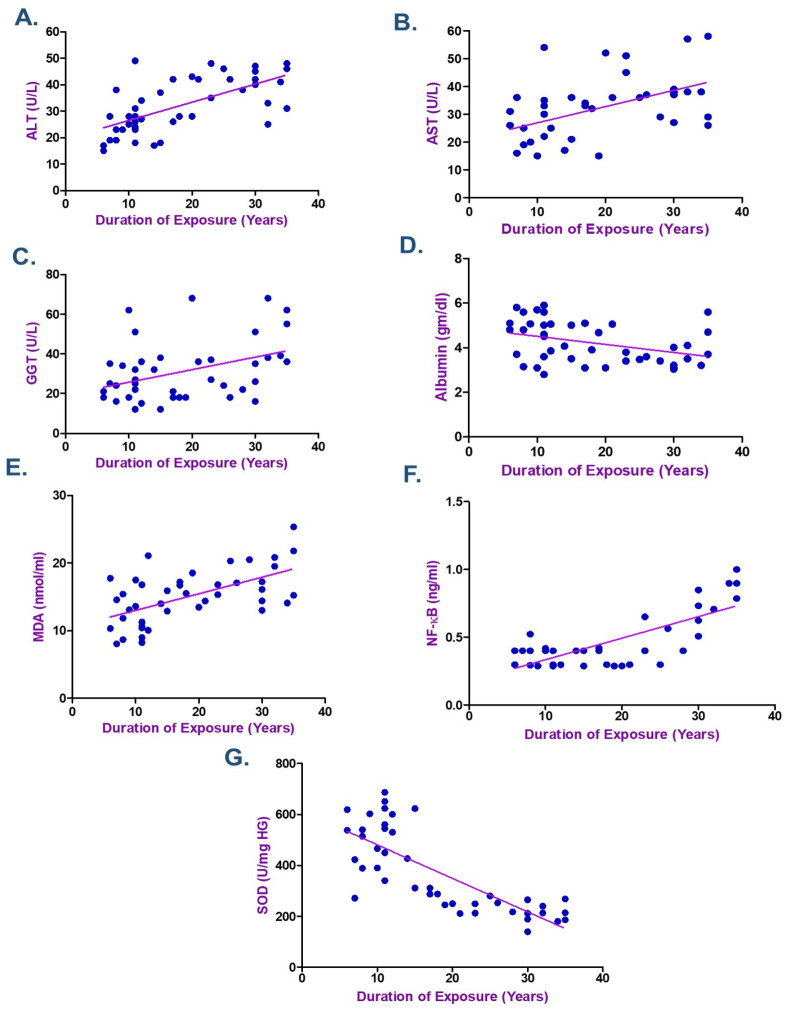
Spearman Correlation between the Duration of Exposure and (**A**) Alanine transaminase (ALT), (**B**) Aspartate transaminase (AST), (**C**) γ-Glutamyl transferase (GGT), (**D**) Albumin, (**E**) Malondialdehyde (MDA), (**F**) Nuclear factor-κB (NF-κB), and (**G**) Superoxide dismutase (SOD) among Electroplating Workers (the line represents the fitted correlation trend, and the dots indicate the individual data points used to calculate the correlation coefficient).

According to the Spearman correlation coefficient in electroplating workers (Table 4 and Table 5), there was a statistically significant positive correlation between the duration of exposure, serum Cr, Ni, and each of MDA, NF-κB, ALT, and AST. The current data showed a negative association between the levels of Cr and Ni and the length of exposure with each of albumin and SOD (*p* < 0.05) as seen in Figure 2, Figure 3 and Figure 4. ALT and AST demonstrated a negative correlation with SOD and Nrf2 gene expression in electroplaters but a positive correlation with the duration of exposure and NF-κB levels.

Additionally, in electroplaters *Ho-1* gene expression was found to be negatively correlated with MDA, serum Ni, age, and duration of exposure (Figure S1). Also, a negative correlation was detected between MDA, serum Cr, Ni, ALT, GGT, age, and duration of exposure with *Nrf2* gene expression (Figure S2). Conversely, the antioxidant enzyme (SOD) had a positive association with *Ho-1* and *Nrf2* gene expression (Table 6).

## 3. Discussion

### 3.1. Clinical Manifestation Prevalence Among Electroplaters

Regarding the prevalence of clinical symptoms among the study participants, electroplaters experienced a higher prevalence of cough and expectoration than nonexposed individuals (30.2% vs. 9.3% and 25.6% vs. 4.7%, respectively). The electroplaters had a higher prevalence of irritating effects, including contact dermatitis (18.6%), sinusitis (16.3%), conjunctivitis (11.6%), and rhinitis (18.6%) (Table 1). These symptoms may be attributed to developing oxygen gas, hydrogen, chromic acid mists, and Ni emissions during electroplating. The findings of this study align with those of other studies demonstrating a statistically significant difference in the prevalence of chest manifestations and allergic reactions between electroplaters and the control group [18,19]. Additionally, Jeyamala et al. found that laborers exposed to Ni and Cr in electroplating businesses in Madurai, South India, had higher rates of allergic bronchitis (17%), nasal irritation (35%), and skin allergic reactions (4.2%) [20]. A study by Were et al. found that employees in Kenya’s welding and leather tanning industries exposed to Ni and Cr had increased rates of wheezing, sneezing bouts, and shortness of breath [21].

### 3.2. Impacts of Occupational Exposure on Serum Cr, Ni, and Liver Function Parameters

Serum levels of Ni (1.3 ± 0.79 µg/L) and Cr (2.47 ± 2 µg/L) were statistically significantly higher in electroplating workers than in the reference group. These results are consistent with the findings of El Safty et al., who examined the genotoxic consequences of combined exposure to Ni and Cr in electroplating workers. The Ni and Cr serum levels in electroplaters ranged from 1.20 to 28.00 µg/L and 0.09 to 7.20 µg/L, respectively (*p* < 0.001) [18]. Our results align with a study by Shaker et al., which found that electroplating workers had statistically significantly higher mean serum Cr and Ni levels than the control group (1.2 ± 0.93 and 1.3 ± 1.02 µg/L vs. 0.4 ± 0.1 and 0.39 ± 0.2 µg/L, respectively) [19].

Renu et al. state that the liver is key in metabolizing heavy metals [6]. The liver can be damaged by heavy metal accumulation [22]. Common indicators of liver damage include total protein (TP), AST, albumin (ALB), and ALT. Serum levels of these indicators rise due to hepatocellular injury, leading to the release of intracellular ALT and AST into the bloodstream [23]. A decrease in TP and ALB levels indicates liver dysfunction, as these proteins are synthesized in the liver [7].

In the present study, electroplating workers’ serum albumin levels significantly declined compared to the controls, while their ALT, AST, and total bilirubin levels were statistically significantly greater than those of the reference group. These results align with a study conducted in Egypt on Ni plating workers, which found that the exposed group had statistically significantly greater levels of Ni, serum AST, and ALT than the unexposed group [24].

According to Fawad and Muhammad, serum TP and ALB levels were considerably lower in Pakistani workers with occupational exposure to Cr (VI) than in those without exposure [25]. Additionally, a study on the occupational exposure of Pakistani women to heavy metals found that workers with higher levels of Cd, Cr, and Ni had lower levels of serum TP and antioxidant enzymes and higher levels of oxidants [26].

Our results are consistent with those of prior experimental studies. AST and ALT levels were elevated as a result of Cr exposure, according to a study by Mohamed et al. [27]. Moreover, the Cr-exposed group exhibited significantly lower TP and ALB levels than the control group in Cr exposure experiments involving fish and rats [28,29]. Cr can damage the liver by interfering with calcium homeostasis, inducing oxidative stress, causing mitochondrial damage, and affecting fatty acid metabolism [30,31].

### 3.3. Oxidative Markers Among Electroplaters

Several investigations have established a link between exposure to Cr and Ni and oxidative stress, which results in inflammation, apoptotic cascades, and liver damage. The present study found that exposure to Ni and Cr significantly decreased the activity of important antioxidant enzymes, particularly GPx (34.73 ± 13.45) and SOD (372.31 ± 162.44), compared to the reference group. Furthermore, we observed that the electroplaters’ MDA levels were significantly higher than those of the control group (15.02 ± 3.99 vs. 5.55 ± 2.38, respectively).

When Cr enters hepatocytes, its reduction generates ROS that produce organic radicals [32]. Cr impedes the respiration chain complexes of mitochondria, resulting in the formation of ROS. Additionally, components derived from Cr reduction actively combine with hydrogen peroxide to generate hydroxyl free radicals [6]. Furthermore, previous studies suggest Ni contributes to ROS production, leading to oxidative stress. This oxidant-antioxidant imbalance linked to Ni is the primary cause of hepatotoxicity, resulting in significant liver damage [8].

The current outcomes agree with a study by Fawad and Muhammad, which investigated biochemical abnormalities and lipid peroxidation in tannery employees exposed to Cr (VI). The study found that blood Cr and MDA levels were significantly elevated (*p* < 0.001) in exposed groups with respect to control groups, while GSH levels were significantly declined (*p* < 0.001) in the exposed groups [25].

### 3.4. Gene Expression and Inflammatory Markers

The body’s antioxidant defense mechanism is activated in response to oxidative injury. A transcription factor known as Nrf2 is essential for the cellular antioxidant system. Nrf2 controls the transcriptional activity of genes that are associated with detoxification and antioxidant formation, enhancing intracellular free radical scavenging and mitigating oxidative stress-induced cellular damage [33]. In the current study, gene expression analysis of the oxidative pathway revealed that electroplating workers occupationally exposed to Cr and Ni had statistically significant downregulation of the *Nrf2* and *Ho-1* genes, while the electroplaters exhibited upregulation of the *Keap-1* gene. *Keap-1* is known to be a negative regulator of *Nrf2* [34].

Disrupting the Nrf2 cascade, a vital cellular defense mechanism against oxidative stress, is a concerning effect of Ni and Cr exposure [35,36]. Several clinical diseases, including liver damage, have been associated with the downregulation of the Nrf2 cascade, essential for regulating the expression of detoxification and antioxidant enzymes. The generation of ROS due to Cr and Ni exposure can lead to oxidative stress and cellular damage [37]. This oxidative stress may, in turn, disrupt the Nrf2 cascade, weakening the cell’s ability to mount an effective defense against the detrimental effects of Cr and Ni exposure.

Previous experimental investigations have documented the downregulation of the *Nrf2* cascade in response to Cr exposure. Research by Han et al. and Lv et al. revealed that exposure to Cr(VI) significantly decreased *Nrf2* expression, with an accompanying increase in inflammation and oxidative damage [10,38]. Furthermore, Yang et al. demonstrated that Cr exposure caused hepatotoxicity by preventing the deacetylation of Nrf2, thereby intensifying inflammation and oxidative stress-mediated apoptosis [12].

In contrast to our results, there was a significant reduction in the expression of *Keap-1* genes and proteins in rodent kidney tissues following exposure to Ni and/or Cr. However, an experimental study by Du et al. demonstrated significantly greater levels of expression of *Nrf2* and its downstream target genes, *Ho-1* and *NQO1* [33]. The Nrf2 antioxidant system can mitigate the effects of oxidative stress by promoting the early production of antioxidant genes. However, if oxidative stress becomes excessively severe, the regulatory capacity of the *Nrf2* pathway may be overwhelmed.

In the present investigation, the electroplaters exhibited a statistically significant increase in the level of the inflammatory marker NF-κB compared to the referent group. Additionally, electroplaters showed statistically significant downregulation of the *miR223* gene compared to the reference group. Although the fundamental mechanisms of Cr-induced hepatotoxicity are still not fully understood, systemic inflammation is believed to play a substantial role [11]. The NF-κB and Nrf2 signaling cascades are believed to work together to regulate the cellular response to stress and inflammation, maintaining the physiological balance of cellular redox status. However, the molecular mechanisms underlying this functional connection remain unclear and appear tissue- and cell-type-specific [39]. According to Saha et al., NF-κB inhibits Nrf2 activation through various mechanisms [40].

It is known that NF-κB is fundamental for controlling a number of inflammatory genes, and excess activation can lead to liver damage [41]. According to Shen et al., Cr(VI) caused liver tissue damage through the NF-κB signaling pathway-mediated inflammatory response [42].

Moreover, recent research suggests that occupational exposure may potentially affect the expression of microRNA, a small non-coding RNA molecule that has been linked to the modulation of inflammatory processes [43]. Liver damage, NF-κB, miR-223, and exposure to Cr and Ni are intricately and multidimensionally related. Cr-induced activation of the NF-κB pathway can lead to the elevation of pro-inflammatory cytokines and oxidative stress, which exacerbates liver injury. Additionally, exposure to Cr can cause dysregulation of *miR-223* expression, which can further aggravate the inflammatory response and impact liver function.

The dysregulation of *miR-223* expression in liver physiology and pathology has been demonstrated in several studies. Dysregulated miR-223 is strongly associated with viral hepatitis, liver inflammation, fibrosis, steatosis, and HCC. Despite its apparent role as a negative regulator of immune modulation and the inflammatory response, the mechanisms by which miR-223 mediates these diverse liver disorders remain unclear [44,45].

Jin et al. demonstrated that treatment with Cr (VI) in animals leads to the infiltration of inflammatory immune cells, liver tissue abnormalities, and hepatocyte vacuolation and necrosis [4]. In a prior investigation, Zhang et al. found that the Cr content in the mice’s liver increased substantially due to dynamic Cr inhalation. They observed hepatocyte enlargement and necrosis, along with inflammatory cell infiltration in the pathological sections. The relationship between chromate exposure and pathological liver damage, in terms of dose, remained unclear [46].

### 3.5. Correlation Between Metals Exposure and Biochemical Indicators

According to the Spearman correlation between the variables examined in electroplating workers (Table 4 and Table 5), a statistically significant positive correlation was observed between the duration of exposure, serum Cr, Ni, and each of MDA, NF-κB, ALT, and AST. Moreover, research showed a negative correlation between Cr and Ni levels, duration of exposure, and albumin, as well as SOD. ALT and AST exhibited a negative correlation with SOD and Nrf2 gene expression in electroplaters but a positive correlation with the duration of exposure and inflammatory marker NF-κB levels. Zhao et al. discovered that Cr levels were inversely correlated with TP (β = −0.57; 95% CI: −0.89, −0.26) and ALB (β = −0.27; 95% CI: −0.47, −0.07) levels in Northeast China, where they investigated the relationship between urine metals and liver function biomarkers [7].

The current findings align with those of Kalahasthi et al., who examined liver function integrity in relation to Ni plating. They found that serum levels of the aminotransferases ALT and AST had a negative correlation with Ni levels, while serum albumin had a positive correlation with urine Ni levels [18].

According to a recent cohort study, 305 employees aged 18 to 93 who had worked for the same company for at least a year and had been exposed to chromate due to their jobs were included. In their investigation, they discovered that chromate exposure was strongly positively correlated with TBIL, DBIL, and ALT. They suggested that systemic inflammation could be a potential mechanism [47]. Moreover, Qayyum et al. discovered that among 150 electroplating workers, the plasma Ni and Cr concentrations were noticeably higher in those who had worked for 10 years [2]. Additionally, previous studies reported a significant dose–response relationship between Cr and ALT [17,48].

Furthermore, this investigation demonstrated a negative correlation between the MDA, serum Cr, Ni, ALT, GGT, duration of employment, and *Nrf2* gene expression. Conversely, SOD was positively correlated with Nrf2 gene expression. Nrf2, a critical regulator, governs the expression of numerous enzymatic antioxidants and contributes to the regulation of ROS levels, including SOD, GPx, and Ho-1. These enzymes are essential for maintaining redox equilibrium and cellular homeostasis [49].

### 3.6. Occupational Exposure Determinants

Inadequate ventilation, extended work hours, direct contact with electroplating baths, and irregular use of personal protective equipment are examples of workplace factors that probably led to workers’ increased exposure levels. The current study facility conducts workplace monitoring once a year to confirm compliance with national safety laws. Data from these monitoring operations consistently show that concentrations of these metals in the surrounding environment and workplace air stay under the permissible limits set by national regulatory agencies [50]. Open doors and roof apertures facilitated natural ventilation, and each workplace was furnished with a ventilation system specifically designed to evacuate gases and particulates directly.

Wearing PPE on a regular basis can guard against dust containing respirable metal particles and decrease direct contact with hazardous substances. Approximately one-third of workers (n = 17, 39.5%) in the current study reported utilizing personal protective equipment (PPE) daily. Chromium, nickel, liver transaminases, and the oxidative marker MDA were all considerably reduced in the current investigation when PPE was used (Table 3). This result was consistent with a study by El Safety et al. that found that using PPE among electroplating workers significantly reduced serum levels of nickel and chromium [18].

### 3.7. Strengths and Limitations

One of this study’s primary strengths is its ability to offer a comprehensive evaluation of the occupational hepatic hazards related to exposure to chromium and nickel by integrating clinical examination, biochemical liver markers, oxidative stress indicators, and gene expression analysis. The reliability of comparisons is improved by including a control group. However, there were certain limitations to the current study. First, rather than providing proof of direct causality, the study’s cross-sectional design limits causal inference. Second, residual confounding may have been introduced by unmeasured co-exposure to additional occupational metals, which could have contributed to the reported oxidative, hepatic, and clinical changes. Furthermore, as nutritional factors may affect systemic levels of chromium, nickel, and antioxidant biomarkers, the lack of food intake assessment is an additional constraint.

## 4. Materials and Methods

### 4.1. Population of Study and Disease Condition

A cross-sectional investigation was performed at a metallurgical factory’s electroplating sector in Ain Helwan, Egypt, from March 2024 to September 2024. The electroplating sector involves worker exposure to Cr, which is used as an electrolyte that generates mist during the plating process. Liquids and powders are transferred to and from plating solutions, and acid granules are weighed and mixed, resulting in additional exposure. Ni compounds, including Ni sulfate and Ni chloride granules and vapors, are frequently encountered by chrome platers, as most Cr plating procedures are linked to Ni plating.

The date of the periodic checkup was communicated verbally and via posters before the study, and participation was offered to all as part of routine medical examinations. The study invited all 74 employees of the electroplating department at the selected factory. All employees had been continuously employed for at least one year, working eight hours per day, five days per week, with occupational exposure to Cr and Ni fumes. After excluding non-responders and applying exclusion criteria, 69 workers remained eligible. Forty-three male non-smoking electroplating workers were selected for inclusion using Microsoft Excel (simple random sampling) based on the estimated sample size. In contrast, the referent group comprised 43 healthy male workers from the same facility. These workers shared comparable socioeconomic and demographic characteristics, such as smoking behaviors, education, and ethnic origin, and had not previously been exposed to heavy metals or chemical agents in the workplace.

#### 4.1.1. Exclusion Criteria

Participants who were obese, smokers, had a history of alcohol consumption, had medical conditions (unmanaged hypertension, diabetes, present or past viral hepatitis, schistosomiasis, or an autoimmune disorder), or were taking medications with potential hepatotoxic effects were excluded.

#### 4.1.2. Sample Size

Eighty-six participants (forty-three per group) were included in this study. The sample size was calculated based on prior data from Kalahasthi and his colleagues to compare AST levels between workers exposed to Ni and those unexposed. The sample size was computed using the G*Power program (version 3.1) with a paired *t*-test family to achieve a 0.71 effect size at 90% power and a 0.05 significance level [51]. All procedures were conducted with the utmost discretion, from sample collection to testing, coding, and result recording. Individuals received copies of the results of the serological tests so they could pursue further evaluation and treatment.

### 4.2. Questionnaire and Clinical Examination

The staff members were provided with a comprehensive explanation of the research plan and examination protocols. To collect data from the reference group and electroplating industry workers, the researchers administered a questionnaire that covered sociodemographic details such as age, smoking habits, place of residence, family history, and current and past medical history. Occupational history, including present type, prior employment, duration of employment, and use of protective equipment. A comprehensive clinical assessment, together with measurements of height and weight and calculation of body mass index (BMI), was performed for all individuals.

### 4.3. Sample Collection and Analysis

In the early morning, a peripheral specimen of blood of 6 mL was collected from each participant by venipuncture using a sterile plastic disposable syringe under stringent aseptic conditions. Before the procedure, the participants were fasting for at least 12 h. The material was then coagulated in a normal Vacutainer tube and centrifuged at 3000× *g* for 10 min to isolate the serum. The serum was then divided into two Eppendorf tubes and preserved at −80 °C. One tube was designated for total RNA extraction, including the quantification of the fold variations in the miR-223 gene through real-time PCR and preserved microRNA extraction, with the mRNAs of *Keap-1*, Nrf2, and Ho-1, while the other tube was allocated for biochemical analysis.

#### 4.3.1. Liver Function Tests

Blood hepatic transaminases (ALT and AST) were measured using traditional clinical laboratory procedures, as well as hepatic cholestasis tests, including gamma-glutamyl transferase (GGT) [52]. Total bilirubin concentrations were measured using the usual diazo reaction method to estimate hepatic clearance [53]. Albumin (ALB) was measured to determine the liver’s synthetic function [54].

#### 4.3.2. Evaluation of Gene Interaction with Chromium

The Comparative Toxigenomics Database (CTD, http://ctdbase.org/; accessed on 25 November 2025) was used to identify Chromium interacting genes. Highly interacting genes were submitted to Cytoscape_v3.10.4 for network visualization (http://www.cytoscape.org/; accessed on 7 December 2025). Chromium was recognized as node hub and connected to different genes (Figure 1).

#### 4.3.3. Measurement of *miR-223* and *Keap-1*, *Nrf2*, and *Ho-1* mRNAs: Fold Changes Isolation of RNA

In accordance with the instructions provided by the manufacturer, the miRNeasy serum/plasma reagent (Catalog Number: 217184) was employed to extract total RNA, including miRNAs, from 200 μL of serum. The purity of the RNA samples was assessed and verified using the NanoDrop ND-1000 spectrophotometer (NanoDrop Technologies, Inc., Wilmington, NC, USA).

Reverse Transcription (RT) and Quantitative Real-Time Polymerase Chain Reaction (qRT-PCR) of miR-223: A total of 10 μL of RT reactions was used to convert extracted RNA to cDNA, following the manufacturer’s procedure, using the miRCURY LNA RT Kit (Catalog Number: 339340) from Qiagen GmbH, Hilden, Germany. The following procedures were executed for RT and cDNA synthesis: Template RNA was introduced to each vial containing the RT master mix; the reverse-transcription master mix was formulated on ice; centrifugation was utilized to achieve gentle mixing; incubation occurred at 42 °C for 60 min, succeeded by enzyme inactivation at 95 °C for 5 min.

A miRCURY LNA SYBR Green PCR kit (Catalog No. 339345) from Qiagen GmbH, Hilden, Germany was used to perform a real-time qPCR test. The protocols for qPCR tests were as follows: (1) A reaction mixture was formulated for a 10 μL per well reaction volume; (2) the real-time cycler was configured to execute a PCR initial activation phase at 95 °C for 2 min, succeeded by a two-step cycling protocol comprising denaturation at 95 °C for 10 s and simultaneous annealing and extension at 56 °C for 60 s. The outcomes were analyzed using the threshold cycle (2^−ΔΔCt^) technique of comparative PCR. The primers used were (1) hsa-miR-223 (Forward: CCACGCTCCGTGTATTTGAC, Reverse: 5′-CCGCACTTGGGGTATTTGAC-3′ [NC_000023.11]); and (2) U6 snRNA (Forward: 5′-GCTTCGGCAGCACATATACTAAAAT-3′, Reverse: 5′-CGCTTCACGAATTTGCGTGTCAT-3′ [NC_000023.11]).

RT and qRT-PCR were conducted for the *Keap-1*, Nrf2, and Ho-1 mRNAs using the ELK Green One-Step qRT-PCR Super Mix kit (Cat. No. EQ 007-02; ELK Biotechnology, Wuhan, China). This kit utilizes a three-step cycling protocol: reverse transcription (1 cycle at 50 °C for 15 min), pre-denaturation (1 cycle at 95 °C for 2 min), and 40 cycles of amplification (annealing at 50–60 °C for 30 s, denaturation at 95 °C for 10 s, and extension at 72 °C for 30 s). Fold changes were measured by qRT-PCR amplification and analysis (StepOne™, Foster City, CA, USA) on an Applied Biosystems platform with software version 3.1. The primer sets were refined for the qRT-PCR test at the annealing temperature. GAPDH was used as a reference gene. The threshold cycle (2^−ΔΔCt^) technique of comparative PCR was used to analyze the data.

The primers used were (1) *Keap-1* (F-CTGGTACATGACAGCACCGT, R-TATCTTGCAAAACGAGGCCC [NC_000019.10]); (2) *Nrf2* (F-CCCCGTGACTAGGCACATTT, R-CTCCGGAGCCCCTAAGTTTG [NC_000002.12]); (3) *Ho-1* (F-TGCAAGGTGAGAGCATCCAG, R-GTACAGCCAGCCTTGATCGT [NC_000022.11]); and (4) *GAPDH* (F-TGTAGGCTCATTTGCAGGGG, R-TCCCATTCCCCAGCTCTCAT [NC_000012.12]).

The quantitative detection of the serum inflammatory marker NF-κB was performed in vitro using the Human NF-κB ELISA kit (Catalog No. MBS450580) which identifies the NF-κB p65 (RelA) subunit. In accordance with the manufacturer’s guidelines, all reagents and standards in the kit were prepared. Spectrophotometric analysis was performed at 450 nm to assess the color variation.

Measuring oxidative biomarkers: Using the colorimetry technique and according to the manufacturer’s guidelines, GPx was measured employing the GPx colorimetric activity kit (Biodiagnostic, Brooklyn, NY, USA, Cat No. GP 2524), and MDA was measured using the MDA content assay kit (SolarBio, Beijing, China, Cat No. BC0020). SOD was measured utilizing the SOD assay kit (SolarBio, Cat No. BC0170).

We measured serum Cr and Ni levels with the Thermo Elemental M6 from Cambridge, England, employing an atomic absorption spectrophotometer outfitted with Zeeman background correction and a graphite furnace. The limits of quantification (LOQ) were 0.40 µg/L for chromium and 0.65 µg/L for nickel, whilst the limits of detection (LOD) were 0.12 µg/L and 0.20 µg/L, respectively. Recovery rates of 94–102% for chromium and 92–101% for nickel were obtained when method accuracy was evaluated using spiking tests with certified control material. Repeated measurements of quality-control samples were used to assess precision; intra-assay CVs of 3.8% (Cr) and 4.5% (Ni) and inter-assay CVs of 5.6% (Cr) and 6.2% (Ni) were found. The concentration of each metal was ascertained by analyzing the standard curves derived from external calibrators for Cr and Ni [55].

### 4.4. Statistical Methods

SPSS version 28 was used for data coding and input. Quantitative data were represented by the mean, standard deviation, median, minimum, and maximum, whilst categorical variables were described by relative frequencies (proportions) and absolute frequencies (number of instances). The unpaired *t*-test was used to compare the groups for regularly distributed quantitative variables, while the non-parametric Mann–Whitney test was utilized for non-normally distributed variables [56]. The chi-square (χ^2^) test was used to compare categorical data. An exact test was used when the anticipated frequency fell below five [57]. Spearman correlation coefficients were used to determine correlations among quantitative variables [58]. *p*-values deemed statistically significant were regarded as those below 0.05.

## 5. Conclusions

This study highlights the potential hepatotoxicity associated with occupational exposure to Cr and Ni. The exposed electroplating workers demonstrated significant change in liver function parameters, which was linked to inflammation, oxidative stress, and downregulation of *Nrf2*/*Ho-1* antioxidant gene expression. These factors are believed to contribute to subsequent liver damage. Collectively, the findings indicate that occupational exposure to Ni and Cr can induce liver affection through inflammation and hepatic oxidative stress, which may be exacerbated by the *Keap-1*/*Nrf2*/*Ho-1* cascade.

Based on the results of this study, we recommend biomonitoring the levels of chromium and nickel regularly, ideally every six months, to enable early detection of excessive absorption or new health effects. Adherence to the minimum ventilation rates advised for electroplating workshops (e.g., local exhaust ventilation capable of attaining at least 100–150 ft^3^/min capture velocity at the plating tanks) is another engineering control that needs to be tightened. In addition, personnel should use standardized personal protective equipment (PPE), including aprons, nonpermeable gloves, and NIOSH-approved respirators, such as P100 or combination P100/acid–gas cartridges. These measures are crucial parts of an all-encompassing occupational health program that aims to lower metal exposure and the health hazards that go along with it, together with regular medical surveillance, environmental monitoring, and worker training. Further longitudinal studies involving a larger cohort of individuals exposed to Ni and Cr in the electroplating industry are necessary to evaluate the effectiveness of antioxidant supplements and/or medications that enhance the Nrf2/Ho-1 pathway in reducing the possible hepatotoxicity brought on by these metals.

## Figures and Tables

**Table 1:** Comparing the prevalence of clinical manifestations among electroplaters and referent participants.

		Electroplating Workers		Referent Group		*p*-Value
		Count	%	Count	%	
Chronic fatigue	Yes	14	32.6%	6	14.0%	0.041 *
	No	29	67.4%	37	86.0%	
Cough	Yes	13	30.2%	4	9.3%	0.015 *
	No	30	69.8%	39	90.7%	
Expectoration	Yes	11	25.6%	2	4.7%	0.007 *
	No	32	74.4%	41	95.3%	
Dyspnea	Yes	6	14%	2	4.7%	0.14
	No	37	86%	41	95.3%	
Allergic rhinitis	Yes	8	18.6%	0	0.0%	0.005 *
	No	35	81.4%	43	100.0%	
Sinusitis	Yes	7	16.3%	2	4.7%	0.156
	No	36	83.7%	41	95.3%	
Conjunctivitis	Yes	5	11.6%	1	2.3%	0.202
	No	38	88.4%	42	97.7%	
Contact dermatitis	Yes	8	18.6%	0	0.0%	0.005 *
	No	35	81.4%	43	100.0%	

* Statistically significant.

**Table 2:** Demographic, anthropometric, and biochemical parameters of electroplating workers and the referent group.

	Electroplating Workers			Referent Group			*p*-Value
	Mean ± SD	Median	Min–Max	Mean ± SD	Median	Min–Max	
Age	40.74 ± 9.86	39	28–59	39.07 ± 7.64	38	29–54	0.381
Duration of exposure (years)	18.26 ± 9.58	15	6–35	16.56 ± 7.36	15	7–33	0.571
BMI (kg/m^2^)	25.25 ± 2.42	25.7	18.8–29.1	25.73 ± 2.61	26.4	18.8–29.1	0.380
Systolic blood pressure	124.88 ± 11.1	125	110–140	123.26 ± 11.01	120	110–145	0.497
Diastolic blood pressure	77.44 ± 8.12	80	60–90	75.58 ± 7.25	75	60–90	0.266
Serum Ni (µg/L)	1.39 ± 0.79	1.30	0.25–2.9	0.51 ± 0.33	0.44	0.02–1.8	<0.001 *
Serum Cr (µg/L)	2.47 ± 2.00	2.02	0.21–7.90	0.45 ± 0.26	0.40	0.10–1.2	<0.001 *
ALT (u/L)	32.16 ± 10.25	31	15–49	26.47 ± 7.11	26.00	15–44	0.013 *
AST (u/L)	31.72 ± 11.36	32	15–58	26.47 ± 8.56	22.00	13–42	0.048 *
GGT (u/L)	30.91 ± 15.15	26	12–68	25 ± 5.07	24.00	16–38	0.278
Total bilirubin (gm/dL)	0.91 ± 0.45	0.94	0.25–1.9	0.67 ± 0.18	0.68	0.36–0.98	0.015 *
Albumin (gm/dL)	4.21 ± 0.93	4.02	2.8–5.9	4.65 ± 0.67	4.70	3.26–5.8	0.013 *
MDA (nmol/mL)	15.02 ± 3.99	15.24	8.04–25.35	5.55 ± 2.38	4.80	2.07–10.2	<0.001 *
SOD (U/mg HG)	372.31 ± 162.44	311	140–687	1241.07 ± 221.41	1297	717–1587	<0.001 *
GPx (mU/mL)	34.73 ± 13.45	34.53	13.98–77.81	109.85 ± 24.85	115.58	58.36–143.89	<0.001 *
NF-κB (ng/mL)	0.46 ± 0.20	0.38	0.29–0.95	0.26 ± 0.04	0.26	0.2–0.36	<0.001 *
miR-223 expression	0.38 ± 0.17	0.36	0.13–0.73	1 ± 0	1.00	1–1	<0.001 *
*Keap-1* expression	1.47 ± 0.23	1.5	1.07–1.86	1 ± 0	1.00	1–1	<0.001 *
Nrf2 expression	0.34 ± 0.19	0.29	0.10–0.73	1 ± 0	1.00	1–1	<0.001 *
Ho-1 expression	0.35 ± 0.21	0.32	0.10–0.83	1 ± 0	1.00	1–1	<0.001 *

* *p* value < 0.05 denotes statistically significant.

**Table 3:** The relation between the use of personal protective equipment and measured biochemical markers among electroplaters.

Use of PPE			
	Yes (n = 17)	No (n = 26)	*p*-Value
Blood nickel (µg/L)	0.72 ± 0.44	1.83 ± 0.65	<0.001 *
Blood chromium (µg/L)	1.07 ± 0.75	3.38 ± 2.04	<0.001 *
ALT (u/L)	26.76 ± 9.45	35.69 ± 99.31	0.005 *
AST (u/L)	23.88 ± 7.01	36.84 ± 10.79	<0.001 *
GGT (u/L)	28.29 ± 12.53	32.61 ± 16.66	0.384
Total bilirubin (gm/dL)	0.93 ± 0.44	0.89 ± 0.46	0.813
Albumin (gm/dL)	4.63 ± 0.98	3.93 ± 0.79	0.021 *
MDA (nmol/mL)	12.52 ± 3.1	16.66 ± 3.69	0.001 *
GPx (mU/mL)	3.68 ± 12.56	34.11 ± 14.21	0.486
NF-kB (ng/mL)	0.35 ± 0.06	0.53 ± 0.23	0.054
*miR-223* expression	0.39 ± 0.18	0.37 ± 0.17	0.921
*Keap-1* expression	1.44 ± 0.21	1.49 ± 0.24	0.455
*Nrf2* expression	0.45 ± 0.17	0.26 ± 0.17	0.002 *
*Ho-1* expression	0.47 ± 0.2	0.27 ± 0.19	0.002 *

* *p* value < 0.05 denotes statistically significant.

**Table 4:** Spearman correlation between blood Ni, Cr, duration of employment, and all biochemical parameters among electroplating workers.

	Serum Ni (µg/L)		Serum Cr (µg/L)		Duration of Exposure (Years)	
	Correlation Coefficient	*p*-Value	Correlation Coefficient	*p*-Value	Correlation Coefficient	*p*-Value
Duration of exposure (years)	0.830	<0.001 *	0.765	<0.001 *		
ALT (u/L)	0.558	<0.001 *	0.626	<0.001 *	0.647	<0.001 *
AST (u/L)	0.590	<0.001 *	0.741	<0.001 *	0.549	<0.001 *
GGT (u/L)	0.411	0.006 *	0.294	0.056	0.390	0.010 *
Total bilirubin (gm/dL)	0.044	0.780	0.191	0.220	0.123	0.434
Albumin (gm/dL)	−0.365	0.016 *	−0.406	0.007 *	−0.352	0.021 *
MDA (nmol/mL)	0.371	0.014 *	0.358	0.018 *	0.535	<0.001 *
GPx (mU/mL)	−0.078	0.621	0.162	0.299	−0.141	0.369
NF-kB (ng/mL)	0.449	0.003 *	0.562	<0.001 *	0.560	<0.001 *
SOD (U/mg HG)	−0.698	<0.001 *	−0.794	<0.001 *	−0.782	<0.001 *

* Statistically significant.

**Table 5:** Spearman correlation of liver enzymes (ALT and AST) with all studied variables among electroplating workers.

	ALT (u/L)		AST (u/L)	
	Correlation Coefficient	*p*-Value	Correlation Coefficient	*p*-Value
Duration of exposure (years)	0.647	<0.001 *	0.549	<0.001 *
MDA (nmol/mL)	0.178	0.253	0.207	0.183
GPx (mU/mL)	0.064	0.683	0.017	0.915
NF-κB (ng/mL)	0.528	<0.001 *	0.421	0.005 *
SOD (U/mg HG)	−0.612	<0.001 *	−0.489	0.001 *
*miR-223* expression	0.005	0.974	−0.068-	0.666
*Keap-1* expression	−0.053	0.735	−0.019	0.906
*Nrf2* expression	−0.390	0.010 *	−0.299	0.051
*Ho-1* expression	−0.293	0.057	−0.258	0.095

* Statistically significant.

**Table 6:** Spearman correlation between gene expression (miR-223, *Keap-1*, Nrf2, and Ho-1) and all studied variables among electroplating workers.

	*miR-223* Expression		*Keap-1* Expression		*Nrf2* Expression		*Ho-1* Expression	
	Correlation Coefficient	*p*-Value	Correlation Coefficient	*p*-Value	Correlation Coefficient	*p*-Value	Correlation Coefficient	*p*-Value
Duration of exposure (years)	−0.094	0.547	0.107	0.495	−0.665	<0.001 *	−0.544	<0.001 *
Serum Ni (µg/L)	−0.001	0.993	0.043	0.785	−0.443	0.003 *	−0.395	0.009 *
Serum Cr (µg/L)	−0.149	0.340	0.003	0.983	−0.433	0.004 *	−0.291	0.058
ALT (u/L)	0.005	0.974	−0.053	0.735	−0.390	0.010 *	−0.293	0.057
AST (u/L)	−0.068	0.666	−0.019	0.906	−0.299	0.051	−0.258	0.095
GGT (u/L)	−0.126	0.420	0.059	0.709	−0.388	0.010 *	−0.121	0.441
Total bilirubin (gm/dL)	−0.211	0.175	0.175	0.261	−0.229	0.140	0.002	0.991
Albumin (gm/dL)	0.225	0.147	0.236	0.128	0.159	0.310	0.209	0.179
MDA (nmol/mL)	−0.167	0.283	0.116	0.459	−0.308	0.044 *	−0.328	0.032 *
GPx (mU/mL)	−0.004	0.982	−0.064	0.684	0.141	0.366	0.219	0.158
NF-κB (ng/mL)	−0.164	0.292	−0.040	0.798	−0.260	0.092	−0.129	0.410
SOD (U/mg HG)	0.164	0.294	0.072	0.645	0.589	<0.001 *	0.480	0.001 *

* Statistically significant.

## Data Availability

The data are not publicly accessible due to ethical concerns; however, it can be obtained upon reasonable request from the corresponding author.

## Supplementary Materials

The following supporting information can be downloaded at: https://www.mdpi.com/article/10.3390/ijms262411954/s1.
