# Supplementary material for: Exploring the Molecular Mechanism of Hepatic Dysfunction Among Workers Exposed to Nickel and Chromium in Electroplating
Source: Int J Mol Sci. 2025 Dec 11;26(24):11954. doi: 10.3390/ijms262411954 (PMC12732974; doi:10.3390/ijms262411954)
Supplement: Supplementary file 1 [file ijms-26-11954-s001.zip › ijms-3962203-supplementary.pdf]

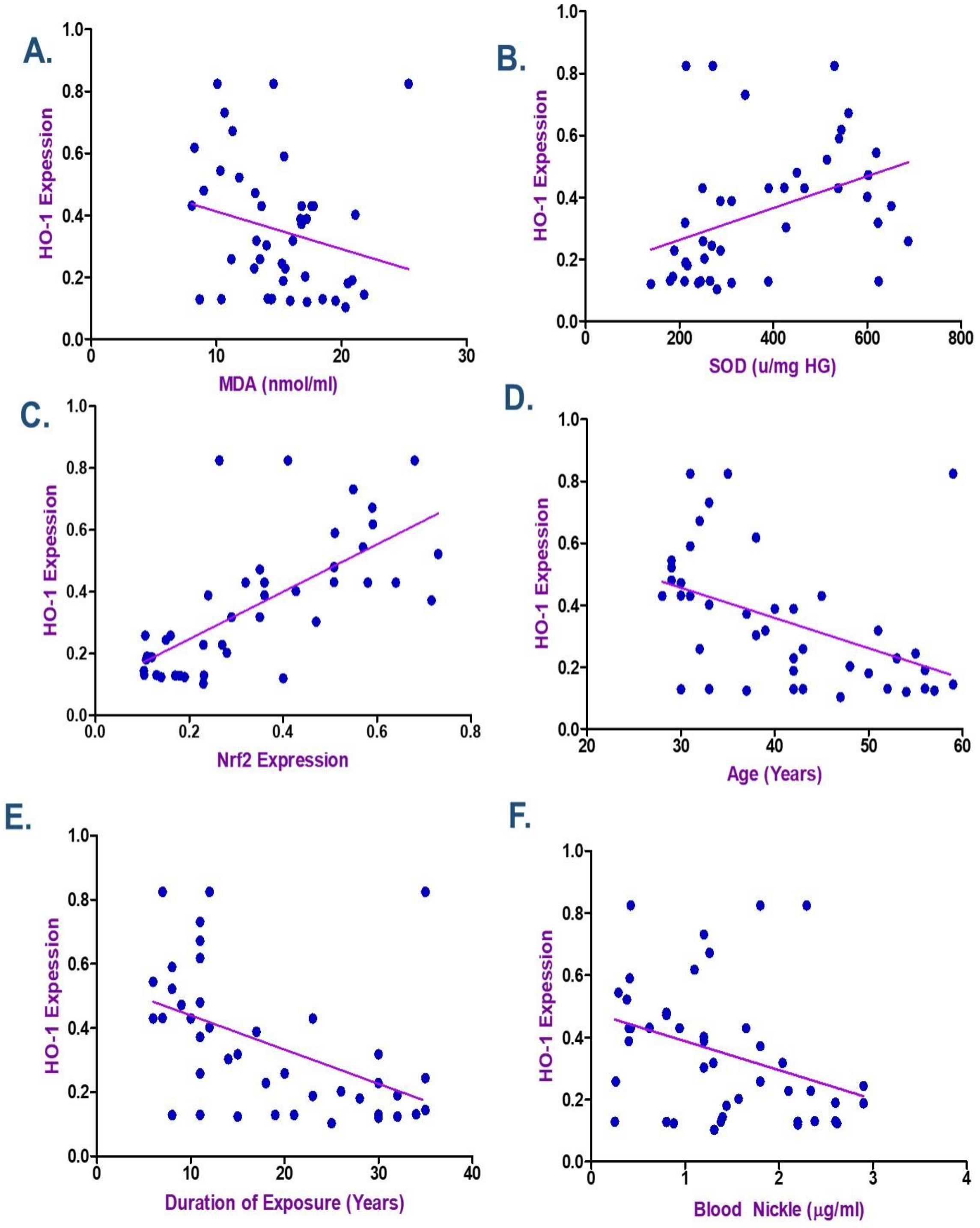

Figure S1. Spearman Correlation between the Heme Oxygenase-1 (*HO-1*) Expression and: (A) Malondialdehyde (MDA), (B) Superoxide dismutase (SOD), (C) *Nrf2* Expression, (D) Age, (E), Duration of exposure, (F) Serum Nickel, among Electroplating Workers.

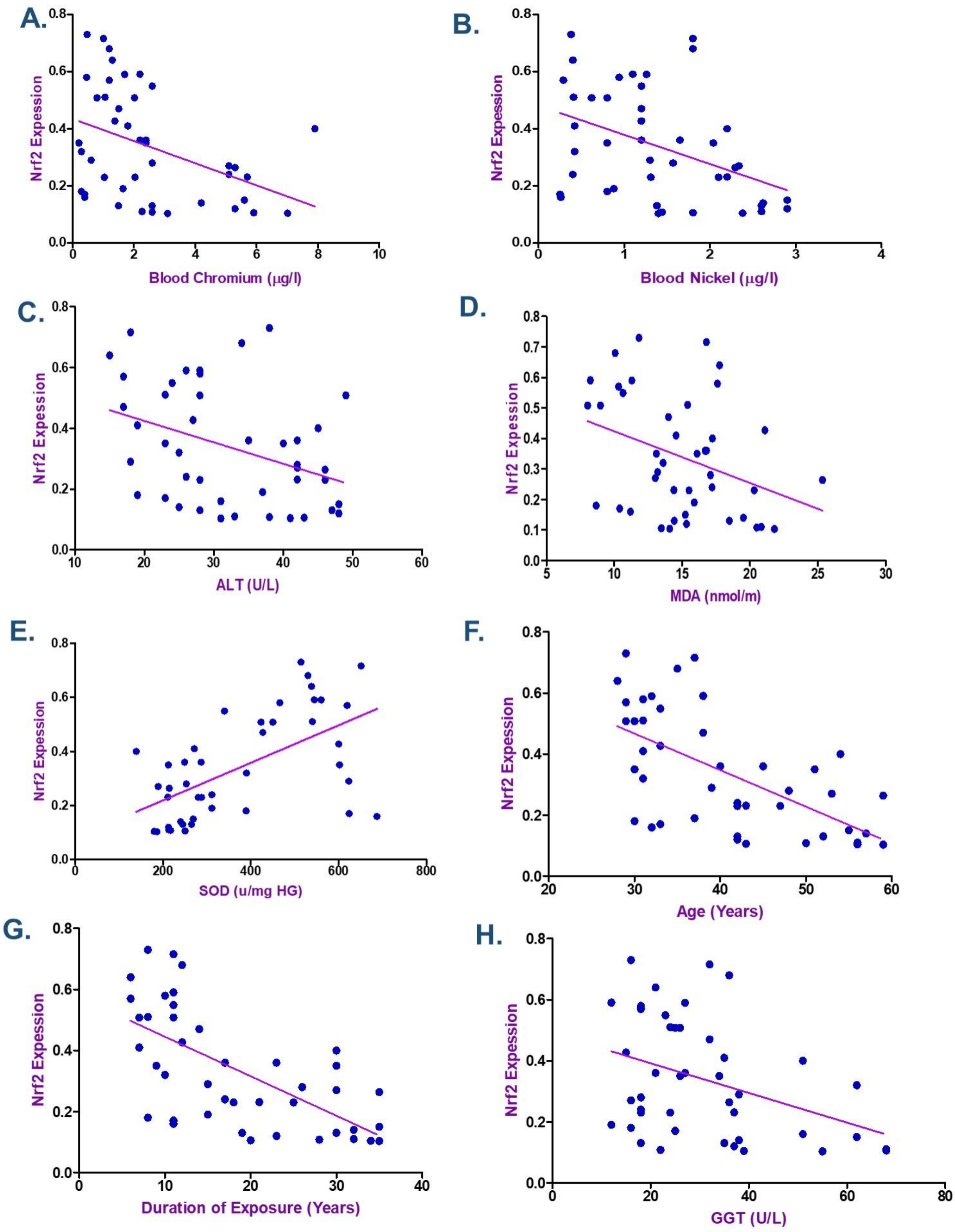

Figure S2. Spearman Correlation between *Nrf2* Expression and: (A) Serum Chromium, (B) Serum Nickel, (C) Alanine transaminase (ALT), (D) Malondialdehyde (MDA), (E) Superoxide dismutase (SOD), (F) Age, (G) Duration of Exposure, (H) γ-Glutamyl transferase (GGT), among Electroplating Workers.
